# The Absence of the Transient Receptor Potential Vanilloid 1 Directly Impacts on the Expression and Localization of the Endocannabinoid System in the Mouse Hippocampus

**DOI:** 10.3389/fnana.2021.645940

**Published:** 2021-02-22

**Authors:** Jon Egaña-Huguet, Itziar Bonilla-Del Río, Sonia M. Gómez-Urquijo, Amaia Mimenza, Miquel Saumell-Esnaola, Leire Borrega-Roman, Gontzal García del Caño, Joan Sallés, Nagore Puente, Inmaculada Gerrikagoitia, Izaskun Elezgarai, Pedro Grandes

**Affiliations:** ^1^Department of Neurosciences, Faculty of Medicine and Nursing, University of the Basque Country UPV/EHU, Leioa, Spain; ^2^Achucarro Basque Center for Neuroscience, Science Park of the University of the Basque Country UPV/EHU, Leioa, Spain; ^3^Department of Pharmacology, Faculty of Pharmacy, University of the Basque Country UPV/EHU, CIBERSAM, Vitoria-Gasteiz, Spain; ^4^Department of Neurosciences, Faculty of Pharmacy, University of the Basque Country UPV/EHU, Vitoria-Gasteiz, Spain

**Keywords:** endovanilloid, endocannabinoid enzymes, dentate gyrus, immunoelectron microscopy, cannabinoid (CB) receptor 1

## Abstract

The transient receptor potential vanilloid 1 (TRPV1) is a non-selective ligand-gated cation channel involved in synaptic transmission, plasticity, and brain pathology. In the hippocampal dentate gyrus, TRPV1 localizes to dendritic spines and dendrites postsynaptic to excitatory synapses in the molecular layer (ML). At these same synapses, the cannabinoid CB_1_ receptor (CB_1_R) activated by exogenous and endogenous cannabinoids localizes to the presynaptic terminals. Hence, as both receptors are activated by endogenous anandamide, co-localize, and mediate long-term depression of the excitatory synaptic transmission at the medial perforant path (MPP) excitatory synapses though by different mechanisms, it is plausible that they might be exerting a reciprocal influence from their opposite synaptic sites. In this anatomical scenario, we tested whether the absence of TRPV1 affects the endocannabinoid system. The results obtained using biochemical techniques and immunoelectron microscopy in a mouse with the genetic deletion of TRPV1 show that the expression and localization of components of the endocannabinoid system, included CB_1_R, change upon the constitutive absence of TRPV1. Thus, the expression of fatty acid amide hydrolase (FAAH) and monoacylglycerol lipase (MAGL) drastically increased in TRPV1^−/−^ whole homogenates. Furthermore, CB_1_R and MAGL decreased and the cannabinoid receptor interacting protein 1a (CRIP1a) increased in TRPV1^−/−^ synaptosomes. Also, CB_1_R positive excitatory terminals increased, the number of excitatory terminals decreased, and CB_1_R particles dropped significantly in inhibitory terminals in the dentate ML of TRPV1^−/−^ mice. In the outer 2/3 ML of the TRPV1^−/−^ mutants, the proportion of CB_1_R particles decreased in dendrites, and increased in excitatory terminals and astrocytes. In the inner 1/3 ML, the proportion of labeling increased in excitatory terminals, neuronal mitochondria, and dendrites. Altogether, these observations indicate the existence of compensatory changes in the endocannabinoid system upon TRPV1 removal, and endorse the importance of the potential functional adaptations derived from the lack of TRPV1 in the mouse brain.

## Introduction

The endocannabinod system (ECS) made up of cannabinoid receptors (CB_1_R, CB_2_R, among others), the main endocannabinoids, 2-arachydonoyl-glycerol (2-AG) and anandamide (AEA), the main synthesizing enzymes for 2-AG (diacylglycerol lipase, DAGL) and AEA (N-acyl phosphatidylethanolamine phospholipase D, NAPE-PLD), and their main degrading enzymes (2-AG: monoacylglycerol lipase, MAGL; AEA: fatty acid amide hydrolase, FAAH) as well as transport proteins, plays an essential role in hippocampal synaptic plasticity (Kano et al., 2009; Castillo et al., 2012; Maccarrone, 2017; Monday et al., 2020). Although the main enzymes for synthesis and degradation of 2-AG and AEA are selectively segregated at either presynaptic or postsynaptic sites (Gulyas et al., 2004; Katona et al., 2006; Yoshida et al., 2006; Blankman et al., 2007; Lafourcade et al., 2007; Starowicz et al., 2007; Puente et al., 2011; Reguero et al., 2011; Suárez et al., 2011) redundant pathways for 2-AG and AEA turnover also exit (for review, see Murataeva et al., 2014). Endocannabinoids regulate synaptic function at both excitatory and inhibitory synapses by retrograde signaling (Kano et al., 2009; Castillo et al., 2012) and also through astrocytes (Navarrete and Araque, 2008, 2010; Han et al., 2012). The CB_1_R is highly localized to inhibitory GABAergic synaptic terminals and preterminals (Kawamura et al., 2006; Katona and Freund, 2012; Steindel et al., 2013; Shu-Jung Hu and Mackie, 2015; Gutiérrez-Rodríguez et al., 2017), in contrast to its limited presence at excitatory glutamatergic terminals (Marsicano et al., 2003; Monory et al., 2006; Puente et al., 2011; Ruehle et al., 2013; Gutiérrez-Rodríguez et al., 2017), astrocytes (Stella, 2010; Han et al., 2012; Metna-Laurent and Marsicano, 2015; Gutiérrez-Rodríguez et al., 2018), and neuronal and astroglial mitochondria (Bénard et al., 2012; Hebert-Chatelain et al., 2014a,b; Gutiérrez-Rodríguez et al., 2018; Jimenez-Blasco et al., 2020). AEA and 2-AG also activate TRPV1 (Zygmunt et al., 1999, 2013; De Petrocellis et al., 2017; Muller et al., 2019) which regulates synaptic transmission and signals pain in the peripheral nervous system (Caterina et al., 2000). It is also in many cells and regions of the central nervous system (Tóth et al., 2005; Cristino et al., 2006) despite the observation of low TRPV1 expression in reporter mouse brain (Cavanaugh et al., 2011). Nonetheless, TRPV1 participates in hefty brain functions, e.g., excitatory and inhibitory synaptic transmission and plasticity, learning and memory, cortical excitability, or fear and anxiety (Marsch et al., 2007; Chávez et al., 2010; Chavez et al., 2014; Puente et al., 2011; Hurtado-Zavala et al., 2017; Bialecki et al., 2020), intervenes in brain neurogenesis, regulates neural proliferation/differentiation rate (Ramírez-Barrantes et al., 2016) and contributes to AEA transport into endothelial cells (Hofmann et al., 2014).

The use of immunoelectron microscopy has revealed localizations from where TRPV1 regulates neural activity. Thus, TRPV1 localized presynaptically to excitatory synaptic terminals in the CA1 hippocampus facilitates AEA-mediated glutamate release (Bialecki et al., 2020). Also, TRPV1 localized postsynaptically to both, inhibitory synapses in the inner 1/3 of the dentate ML (Canduela et al., 2015) regulates GABAergic synaptic transmission (Chavez et al., 2014), and to excitatory synapses in the outer 2/3 of the ML (Puente et al., 2015) where AEA triggers TRPV1-dependent and CB_1_R-independent long-term depression of the excitatory synaptic transmission (eLTD) at the MPP synapses (Chávez et al., 2010). Interestingly, at these same excitatory synapses, MPP stimulation (10 Hz, 10 min) triggered a group I metabotropic glutamate receptor-dependent and CB_1_R-mediated eLTD that required intracellular calcium and 2-AG synthesis (Peñasco et al., 2019). So, the question raises as whether both receptors would influence each other by acting from opposite loci of the same synapse. In this sense, the existence of interactions between both the endocannabinoid and endovanilloid system has been suggested (Starowicz et al., 2007). AEA increase reduces 2-AG effect on presynaptic CB1Rs through postsynaptic TRPV1 resulting in short-term plasticity regulation (Maccarrone et al., 2008; Musella et al., 2010; Lee et al., 2015). Genetic deletion of endocannabinoid system components leads to compensatory changes in TRPV1; for instance, TRPV1 expression decreases in dentate gyrus and increases in the cerebellar granule cell layer in mice lacking CB_1_R (Cristino et al., 2006). Also, CB_1_R and TRPV1 expressed in the same cell mediate opposite effects on intracellular calcium levels (Szallasi and Di Marzo, 2000), glutamate release (Marinelli et al., 2003) or excitatory and inhibitory neurotransmission (Tahmasebi et al., 2015). Altogether, these previous findings endorse reciprocal and complex bidirectional interactions between CB_1_R and TRPV1 expressed at the same synapse (Zádor and Wollemann, 2015).

We sought in this study compensatory mechanisms in the endocannabinoid system in a mouse model carrying the genetic deletion of TRPV1 (TRPV1^−/−^), which might eventually have an impact on neural information processing. Hence, biochemical and immunohistochemical tools were used to explore the overall expression patterns of some key endocannabinoid system components (DAGL, MAGL, NAPE-PLD, FAAH). Also, immunoelectron microscopy was applied to assess CB_1_R rearrangements in the TRPV1^−/−^ hippocampal dentate gyrus where both receptors are involved in mechanistically distinct eLTD at the MPP synapses (Chávez et al., 2010; Peñasco et al., 2019). The results show that the expression and localization of some components of the endocannabinoid system change upon the constitutive absence of TRPV1.

## Materials and Methods

### Animal Procedures

All protocols were approved by the Committee of Ethics for Animal Welfare of the University of the Basque Country (CEEA/M20/2015/105; CEIAB/M30/2015/106) and were in accordance to the European Communities Council Directive of 22nd September 2010 (2010/63/EU) and Spanish regulations (Real Decreto 53/2013, BOE 08-02-2013). All efforts were made to minimize pain and suffering and to reduce the number of animals used. Eight week-old-male TRPV1^−/−^ mice and their wild type (WT) littermates (TRPV1+/+) were used (*n* = 18 each). The TRPV1^−/−^ mice (C57BL/6 J background; Caterina et al., 2000) were derived from heterozygous breeding pairs generated by crossing of B6.129X1-*Trpv1*^*tm*1*Jul*^/J mice (The Jackson Laboratory, Bar Harbor, ME) with C57BL/6 j mice (Janvier Labs) at the General Animal Unit Service of the University of the Basque Country (UPV/EHU). The mice used were genotyped in the Genomics and Proteomics Unit of the University of the Basque Country (UPV/EHU).

Mice were housed in pairs or groups of maximum three littermates in standard Plexiglas cages (17 × 14.3 × 36.3 cm) and before experiments were conducted, they were allowed to acclimate to the environment for at least 1 week. They were maintained at standard conditions with food and tap water *ad libitum* throughout all experiments in a room with constant temperature (22°C), and kept in a 12:12 h light/dark cycle with lights off at 9:00 p.m.

### Tissue Preservation

The TRPV1^−/−^ and WT mice were deeply anesthetized by intraperitoneal administration of a mixture of ketamine/xilacine (80/10 mg/kg body weight). They were transcardially perfused at room temperature (RT) with phosphate buffered saline (0.1 M PBS, pH 7.4) for 20 s, followed by the iced-cooled fixative solution made up of 4% formaldehyde (freshly depolymerized from paraformaldehyde), 0.2% picric acid, and 0.1% glutaraldehyde in 0.1 M phosphate buffer (PB, pH 7.4) for 10–15 min. Then, brains were carefully removed from the skull and post-fixed in the fixative solution for 1 week at 4°C followed by their storage in 1:10 fixative solution diluted in 0.1 M PB with 0.025% sodium azide at 4°C until use.

### Immunohistochemistry for Light Microscopy

The procedure was published elsewhere (Gutiérrez-Rodríguez et al., 2017). Briefly, 50 μm-thick brain coronal vibratome sections were cut and collected in 0.1 M PB at RT. Hippocampal sections were preincubated in 10% horse normal serum (HNS), 0.1% sodium azide, and 0.5% Triton X-100 in Tris-HCl-buffered saline (TBS) (pH 7.4) for 30 min at RT, and then incubated with one of the following primary polyclonal antibodies: goat anti-CB_1_R (2 μg/ml; Frontier Institute co., ltd; CB1-Go-Af450; RRID: AB_2571591); rabbit anti-MAGL (2 μg/ml; Frontier Institute co, ltd; MGL-Rb-Af200; RRID: AB_2571798); rabbit anti-DAGLα (2 μg/ml; Frontier Institute co, ltd; DGLa-Rb-Af380; RRID: AB_2571691); guinea pig anti-NAPE-PLD (4 μg/ml; Frontier Institute co, ltd; NAPE-PLD-Gp-Af720; RRID: AB_2571806) or rabbit anti-FAAH (1 μg/ml; Cayman Chemical; 101600-1; RRID: AB_327842), on a shaker for 2 days at 4°C. After several washes in 1% HNS and 0.5% Triton X-100 in TBS, the respective hippocampal samples were incubated with the corresponding biotinylated horse anti-goat IgG (1:200; Cat# BA-9500; RRID: AB_2336123; Vector Laboratories, Burlingame, CA), goat anti-rabbit IgG (1:200; Cat# BA-1000; RRID: AB_2313606; Vector Laboratories, Burlingame, CA), goat anti-guinea pig IgG (1:200; Cat# bs-03586; RRID: AB_10860553; Bioss, USA) for 1 h on a shaker at RT, washed in the solution described above and processed for the avidin-biotin peroxidase complex method (ABC; 1:50; Elite; Cat#PK-6100, Vector Laboratories, Burlingame, CA; RRID: AB_2336819). Hence, sections were incubated in the avidin-biotin complex prepared in washing solution (1:50) for 1 h at RT. Then, they were washed and incubated with 0.05% diaminobenzidine (DAB) in 0.1 M PB with 0.5% Triton X-100 and 0.01% hydrogen peroxide, for 5 min at RT. Finally, tissue was mounted, dehydrated in graded alcohols (50, 70, 96, 100%) to xylol and coverslipped with DPX. Sections were observed and photographed with a Zeiss Axiophot light microscope. All micrographs were taken at the same light intensity and exposure time. Adjustments in contrast and brightness were made to the figures in Adobe Photoshop (CS3, Adobe Systems; RRID: SCR_014199).

### CB_1_R Immunoelectron Microscopy

The procedure has already been described in detail elsewhere (Puente et al., 2019). Briefly, hippocampal sections were pre-incubated in a blocking solution of 10% HNS, 0.1% sodium azide, and 0.02% saponine prepared in TBS (pH 7.4) for 30 min at RT. Then hippocampal sections were incubated with a goat anti-CB_1_R antibody (2 μg/ml, #CB1-Go-Af450, Frontier Science Co.; RRID: AB_257130) in 10% HNS/TBS containing 0.1% sodium azide and 0.004% saponine on a shaker for 2 days at 4°C. After several washes in 1% HNS/TBS, tissue sections were incubated with a 1.4 nm gold-labeled rabbit anti-goat IgG (Fab' fragment, 1:100, Nanoprobes Inc., Yaphank, NY, USA Cat#2004; RRID: AB_2631182) in 1% HNS/TBS with 0.004% saponine on a shaker for 4 h at RT. Thereafter, hippocampal sections were washed in 1% HNS/TBS overnight at 4°C, postfixed in 1% glutaraldehyde in TBS for 10 min and washed in double-distilled water. Then, gold particles were silver-intensified with a HQ Silver kit (Nanoprobes Inc., Yaphank, NY, USA; Cat#2012) for ~12 min in the dark and washed in 0.1 M PB. Stained sections were osmicated (1% OsO4 (v/v) in 0.1 M PB, 20 min; Electron Microscopy Sciences; Cat#19150), dehydrated in graded alcohols to propylene oxide and plastic-embedded in Epon resin 812. Ultrathin sections (50 nm-thick) were collected on nickel mesh grids, stained with 2.5% lead citrate for 20 min and examined in an electron microscope (Philips EM208S). Tissue preparations were photographed by using a digital camera (Digital Morada Camera, Olympus) coupled to the electron microscope. Adjustments in contrast and brightness were made to the figures using Adobe Photoshop (CS3, Adobe Systems; RRID: SCR_014199).

### Double CB_1_R and Glial Fibrillary Acidic Protein (GFAP) Immunoelectron Microscopy

Co-labeling experiments were performed as described (Puente et al., 2019). The first steps were shared with the single pre-embedding immunogold method. Then, the hippocampal sections were simultaneously incubated with the goat anti-CB_1_R antibody (2 μg/ml, #CB1-Go-Af450, Frontier Science Co.; RRID: AB_257130) and a mouse anti-GFAP antibody (20 ng/ml; G3893; Sigma-Aldrich, mouse monoclonal; RRID: AB_257130) in 10% HNS/TBS with 0.1% sodium azide and 0.004% saponin on a shaker for 2 days at 4°C. After several washes in 1% HNS/TBS, tissue sections were incubated with both 1.4 nm gold-labeled rabbit anti-goat IgG (Fab' fragment, 1:100, Nanoprobes Inc., Yaphank, NY, USA) for the localization of CB_1_R and a biotinylated horse anti-mouse IgG (1:200 Vector Labs, Cat#BA-2000; RRID: AB_2313581) for the localization of GFAP, diluted in 1% HNS/TBS with 0.004% saponin on a shaker for 4 h at RT. Then, sections were incubated in avidin-biotin peroxidase complex (ABC) prepared in 1% HNS/TBS for 1.5 h at RT. They were subsequently washed in 1% HNS/TBS overnight at 4°C and postfixed in 1% glutaraldehyde in TBS for 10 min at RT. Following several washes in double-distilled water, gold particles were silver intensified with an HQ Silver kit (Nanoprobes Inc., Cat#2012) for ~12 min in the dark and washed in 0.1 M PB, pH 7.4. Then, the tissue was incubated in 0.05% DAB (Sigma-Aldrich, Cat#D5637; RRID: AB_2336819) and 0.01% hydrogen peroxide prepared in 0.1 M PB for 3 min. Labeled sections were osmicated (1% osmium tetroxide, Electron Microscopy Sciences, Cat#19150) in 0.1 M PB, pH 7.4, 20 min, dehydrated in graded alcohols to propylene oxide, and plastic-embedded in Epon resin 812. Ultrathin sections (50 nm-thick) were collected on nickel mesh grids, counterstained with 2.5% lead citrate for 20 min and examined with an electron microscope (JEOL JEM 1400 Plus). Tissue samples were imaged using a digital camera (sCMOS). Figures were created with Adobe Photoshop (CS3, Adobe Systems; RRID: SCR_014199).

### Semi-quantification Analysis

Hippocampal sections from TRPV1^−/−^ (*n* = 3) and WT mice (*n* = 3) were visualized under a light microscope in order to select portions of the inner 1/3 (hilar mossy cell axon terminal synapses) and outer 2/3 of the dentate ML (perforant path synapses) with good, reproducible immunolabeling and well-preserved ultrastructure. All electron micrographs were taken at ×22,000 magnification and showed similar labeling intensity, indicating that the selected areas were at the same depth. Furthermore, only ultrathin sections within the first 1.5 μm from the surface of the tissue block were examined to avoid false negatives. Metal particles placed on membranes were counted. Positive labeling was considered if at least one immunoparticle was over the membrane or within ~30 nm of it. Image-J (FIJI) (NIH, USA; RRID: SCR_003070) was used to measure the membrane length. Sampling was carefully and accurately carried out in the same way for all the animals studied, and experimenters were blinded to the subject during CB_1_R quantification.

Synaptic terminals were identified by ultrastructural features. Thus, asymmetric excitatory synapses showed typical presynaptic terminals containing abundant clear and spherical synaptic vesicles, and thick postsynaptic densities mostly on dendritic spines. Inhibitory synapses had presynaptic terminals with pleomorphic synaptic vesicles forming symmetric contacts with postsynaptic dendrites. Astrocytes were identified by GFAP immunoreaction product inside their cell bodies and processes. The analysis was done over 1,910 synapses, 4,574 mitochondria and 549 astrocytic profiles in TRPV1^−/−^; 2,177 synapses, 5,565 mitochondria and 413 astrocytic profiles in WT.

Image-J (FIJI) (NIH, USA; RRID: SCR_003070) was used to measure the following parameters: percentage of CB_1_R-positive terminals, mitochondria, and astrocytic profiles; density of CB_1_R particles in terminal and astrocytic membranes (particles/μm membrane); terminal perimeter; number of terminals, mitochondria and astrocytic profiles; and proportion of CB_1_R particles in each compartment vs. total CB_1_R labeling. All values were shown as mean ± S.E.M. using a statistical software package (GraphPad Prism 5; GraphPad Software; RRID: SCR_002798). The normality test (Kolmogorov-Smirnov) was always applied before running statistical tests. Sample uniformity was assessed by one-way ANOVA or Kruskal-Wallis multiple comparison test. Data from each group (*n* = 3) were pooled since no significant differences were detected among mice (*p* > 0.05). Finally, data were analyzed by parametric and non-parametric tests (Unpaired *t*-test or Man-Whitney test). Values of *p* < 0.05 were considered statistically significant.

### Whole Hippocampal Homogenates

Mice were deeply anesthetized by inhalation of isoflurane (2–4%) before decapitation. Hippocampi from both hemispheres were dissected and manually homogenized with a plastic stick in a lysis buffer composed of 10 mM PB (pH 7.4), 5 mM ethyleneglycol-bis (2-aminoethylether)- N,N,N′,N′ tetraacetic acid, 5 mM ethylene-diamine-tetra-acetic acid, 1 mM dithiotreitol, and a protease inhibitor cocktail (Ref. P-8340, Sigma-Aldrich). Thereafter, samples were kept 30 min on ice and centrifuged for 15 min at 16,000 g. The resulting supernatant was used as soluble protein extract. Protein concentrations were estimated using Bio-Rad Protein Assay reagent (Ref. 500-0006, Bio-Rad Laboratories SA).

### Hippocampal Membrane Preparation

Hippocampal sections from TRPV1^−/−^ and WT were thawed in ice-cold 20 mM Tris-HCl, pH 7.4, containing 1 mM EGTA (Tris/EGTA buffer), and then homogenized in 20 times the volume of the same hypotonic buffer using a glass homogenizer. Cell debris was discarded by centrifugation at 1,000 g (10 min, 4°C) and then membranes were obtained by centrifugation at 40,000 g (30 min, 4°C). Finally, the pellet was re-suspended and re-centrifuged under the same conditions. Membranes were aliquoted in microcentrifuge tubes, centrifuged again (40,000 g, 30 min, 4°C) and the pellets were stored at −75°C prior to use. Protein content was determined using the Bio-Rad dye reagent with bovine γ-globulin as a standard.

### Protein Determination

Hippocampal extracts from TRPV1^−/−^ and WT were boiled in urea-denaturing buffer [20 mM Tris-HCl, pH 8.0, 12% glycerol, 12% urea, 5% dithiothreitol, 2% sodium dodecyl sulfate (SDS), 0.01% bromophenol blue] for 5 min. Increasing amounts of denatured proteins were resolved by electrophoresis on SDS–polyacrylamide (SDS–PAGE) gels (10%) using the Mini Protean II gel apparatus (Bio-Rad, Hercules, CA, USA). Proteins were transferred to polyvinylidene fluoride (PVDF) membranes (Amersham Bioscience, Buckinghamshire, UK) using the Mini TransBlot transfer unit (Bio-Rad, Hercules, CA, USA) at 90 V constant voltage for 1 h at 4°C. Blots were blocked in 5% non-fat dry milk/PBS containing 0.5% BSA and 0.2% Tween for 1 h, and incubated overnight at 4°C with specific antibodies against CB_1_R (0.2 μg/ml; Frontier Science Co., rabbit polyclonal; CB1-Rb-Af380; RRID: AB_2571591), MAGL (0.2 μg/ml; Frontier Science Co., rabbit polyclonal; MGL-Rb-Af200; RRID: AB_2571798), DAGL (0.2 μg/ml; Frontier Science Co., rabbit polyclonal; DGLa-Rb-Af380; RRID: AB_2571691), NAPE-PLD (0.2 μg/ml; Frontier Science Co., guinea pig polyclonal; NAPE-PLD-Gp-Af720; RRID: AB_2571806), FAAH (0.2 μg/ml; Cayman Chemical, rabbit polyclonal; 101600-1; RRID: AB_327842), and CRIP1a (0.4 ng/μl; Santa Cruz Biotechnology, rabbit polyclonal; sc-137401; RRID: AB_10709018). Blots were washed and incubated with horseradish peroxidase (HRP) conjugated secondary antibodies; goat anti-rabbit IgG HRP (1 ng/ml; Cell Signaling Technology; 7074; RRID: AB_2099233) and goat anti-guinea pig IgG HRP (1 ng/ml; Bioss; bs-0358G; RRID: AB_10860553) diluted to 1:10,000 in blocking buffer for 2 h at RT. After the enhanced chemiluminiscence detection (Santa Cruz) in an Autochemi-UVP Bioimaging System, bands were quantified with Image-J (FIJI) (NIH, USA; RRID: SCR_003070).

### Western Blotting of Hippocampal Synaptosomes

Hippocampal synaptosomes were prepared as previously described (Garro et al., 2001). TRPV1^−/−^ and WT mice were anesthetized with isoflurane and decapitated; brains were removed and placed on ice-cold 0.32 M sucrose, pH 7.4, containing 80 mM Na2HPO4 and 20 mM NaH2PO4 (sucrose phosphate buffer) with protease inhibitors (Iodoacetamide 50 μM, PMSF 1 mM). The hippocampal tissue was minced and homogenized in 10 volumes of sucrose/phosphate buffer using a motor-driven Potter Teflon glass homogenizer (motor speed 800 rpm; 10 up and down strokes; mortar cooled in an ice-water mixture throughout). The homogenate was centrifuged at 1,000 × g for 10 min and obtained pellet (P1) was re-suspended and pelleted. The supernatants (S1 + S1') were pelleted at 15,000 × g (P2) and re-suspended in the homogenization buffer to a final volume of 16 ml. This P2 fraction is a mixture of myelin fragments, synaptosomes and free mitochondria. The suspension was layered directly onto tubes containing 8 ml 1.2 M sucrose phosphate buffer, and centrifuged at 180,000 × g for 20 min. The material retained at the gradient interface (synaptosome + myelin + microsome) was carefully collected with a Pasteur-pipette and diluted with ice-cold 0.32 M sucrose/phosphate buffer to a final volume of 16 ml. The diluted suspension was then layered onto 8 ml of 0.8 M sucrose phosphate buffer, and centrifuged as described above. The obtained pellet was re-suspended in ice-cold phosphate buffer, pH 7.5 and aliquoted in microcentrifuge tubes. Aliquots were then centrifuged at 40,000 × g for 30 min, the supernatants were aspirated and the pellets corresponding to the nerve terminal membranes were stored at −80°C. Protein content was determined using the Bio-Rad dye reagent with bovine γ-globulin as standard.

For western blotting, hippocampal synaptosome fractions were boiled in urea-denaturing buffer [20 mM Tris-HCl, pH 8.0, 12% glycerol, 12% Urea, 5% dithiothreitol, 2% sodium dodecyl sulfate (SDS), 0.01% bromophenol blue] for 5 min. Denaturized proteins were resolved by electrophoresis on SDS–polyacrylamide (SDS-PAGE) gels and transferred to nitrocellulose or PVDF membranes at 30 V constant voltage overnight at 4°C. Blots were blocked in 5% non-fat dry milk/phosphate buffered saline containing 0.5% BSA and 0.1% Tween for 1 h, and incubated with the antibodies overnight at 4°C. Blots were washed and incubated with specific HRP conjugated secondary antibodies diluted in blocking buffer for 1.5 h at RT. Immunoreactive bands were incubated with the ECL system according to the manufacturer instructions. In these experiments, differences between the relative expressions of proteins were analyzed by regression line slopes comparison method by a statistical software package (GraphPad Prism, GraphPad Software Inc, San Diego, USA).

## Results

### Cannabinoid Immunohistochemistry in TRPV1^−/−^ Hippocampus

**Figure 1:**
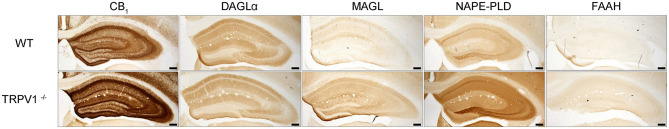
Immunoperoxidase method for light microscopy. Expression patterns of CB_1_R and endocannabinoid enzymes in WT and TRPV1^−/−^ mice hippocampi. MAGL, NAPE-PLD, and FAAH immunoreactivity increases and DAGLα decreases in TRPV1^−/−^. CB_1_R immunoreactivity increases overall TRPV1^−/−^ hippocampus. Scale bars: 500 μm.

The patterns of CB_1_R and the main enzymes for synthesis and degradation of 2-AG and AEA were studied in TRPV1^−/−^ (Figure 1). An increase in MAGL, FAAH, and NAPE-PLD, and a slight decrease in DAGLα immunoreactivity was observed in TRPV1^−/−^ vs. WT. Though MAGL and FAAH immunostainings were faint in both TRPV1^−/−^ and WT hippocampus, MAGL immunoreactivity increased more than FAAH and NAPE-PLD, especially in the hilus and CA3 stratum lucidum. Noticeably, CB_1_R staining increased overall TRPV1^−/−^ hippocampus, but particularly stronger was in CA3 stratum radiatum, CA1 pyramidal cell layer and dentate ML. In the latter, CB_1_R immunoreactivity was more intense in a fiber meshwork in the inner 1/3 of the layer and weaker but yet more conspicuous than in WT, in fibrous profiles distributed in the outer 2/3 ML (Figure 1).

### Expression of ECS Proteins in Whole Hippocampal Homogenates and Synaptosomes of TRPV1^−/−^ Hippocampus

**Figure 2:**
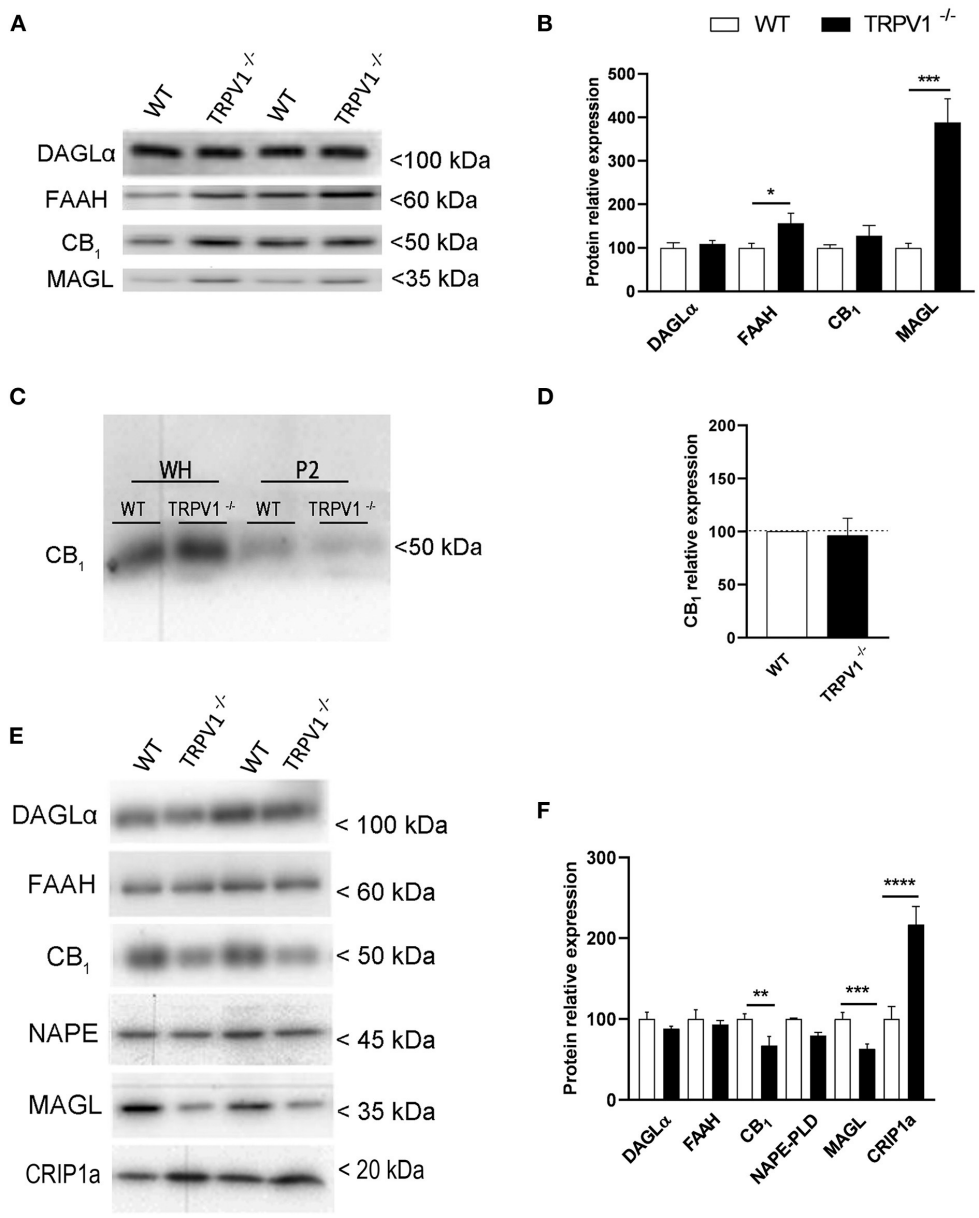
**(A,B)** Immunoblot and relative expression of CB_1_R and endocannabinoid enzymes in hippocampal whole homogenates from WT and TRPV1^−/−^ mice. MAGL and FAAH increase significantly in TRPV1^−/−^. Data were analyzed by means of non-parametric or parametric tests (Mann–Whitney *U*-test or Student's *t*-test). **(C)** Immunoblot of CB_1_R in whole homogenates and P2 extracts of raw membranes from WT and TRPV1^−/−^ mice hippocampi. **(D)** Relative expression of CB_1_R in P2 extracts from WT and TRPV1^−/−^. Not significant changes are detected in TRPV1^−/−^. Student's *t*-test. **(E,F)** Immunoblot and relative expression of CB_1_R, endocannabinoid enzymes, and CB_1_R interacting protein 1a in hippocampal synaptosomes from WT and TRPV1^−/−^ mice. CB_1_R and MAGL decrease but CRIP1a increases significantly in TRPV1^−/−^. Fisher's exact test. *p* > 0.05; *p* < 0.05*; *p* <0.005**; *p*< 0.001***. Data are expressed as mean ± S.E.M.

The expression of CB_1_R, DAGLα, FAAH, and MAGL was investigated in whole homogenate extracts obtained from TRPV1^−/−^ and WT hippocampi (Figure 2A). The ~27.5% increase observed for CB_1_R in TRPV1^−/−^ was not statistically significant (127.5 ± 23.83% vs. control 100%; *p* = 0.301 ns; *n* = 8; Figure 2B). However, FAAH significantly increased (~56%) (156.3 ± 23.52 vs. control 100%; *p* = 0.0467^*^, *n* = 8; Figure 2B) and strikingly did MAGL in TRPV1^−/−^ (~288%) relative to WT (388.1 ± 54.7% vs. control 100%; *p* = 0.0001^***^, *n* = 8; Figure 2B). DAGLα remained unchanged (109.5 ± 7.5% vs. control 100%; *p* = 0.2476 ns; *n* = 2; Figure 2B).

The CB_1_R compartmentalization was studied in homogenates purified to P2 fractions (Figure 2C). Again, receptor expression did not vary significantly between TRPV1^−/−^ and WT (96.18 ± 16.38% vs. control 100%; *p* = 0.8213 ns; Figure 2D). However, synaptosomal fractions extracted from TRPV1^−/−^ homogenates (Figure 2E) revealed a significant ~45% decrease in CB_1_R (3.21 ± 0.82; WT: 5.87 ± 0.50; *p* = 0.0092^**^; Figure 2F) and ~42% decrease in MAGL (3.76 ± 1.16; WT: 6.51 ± 1.38; *p* = 0.0431^*^; Figure 2F). The reduction was not significant for NAPE-PLD (TRPV1^−/−^: 3.49 ± 1.26; WT: 4.83 ± 0.80; *p* = 0.4186 ns; Figure 2F). In contrast, a significant increase in CRIP1a was detected in TRPV1^−/−^ (18.41 ± 1.89; WT: 8.48 ± 1.32; *p* = 0.0006^***^; Figure 2F). Finally, the expression of DAGLα (TRPV1^−/−^: 5.72 ± 0.20; WT: 6.49 ± 0.5544; *p* = 0.31 ns; Figure 2F) and FAAH did not change in TRPV1^−/−^ (TRPV1^−/−^: 5.97 ± 0.32; WT: 6.41 ± 0.72; *p* = 0.607 ns; Figure 2F).

### Cellular and Subcellular Localization of CB_1_R in the Outer 2/3 Dentate ML of TRPV1^−/−^

**Figure 3:**
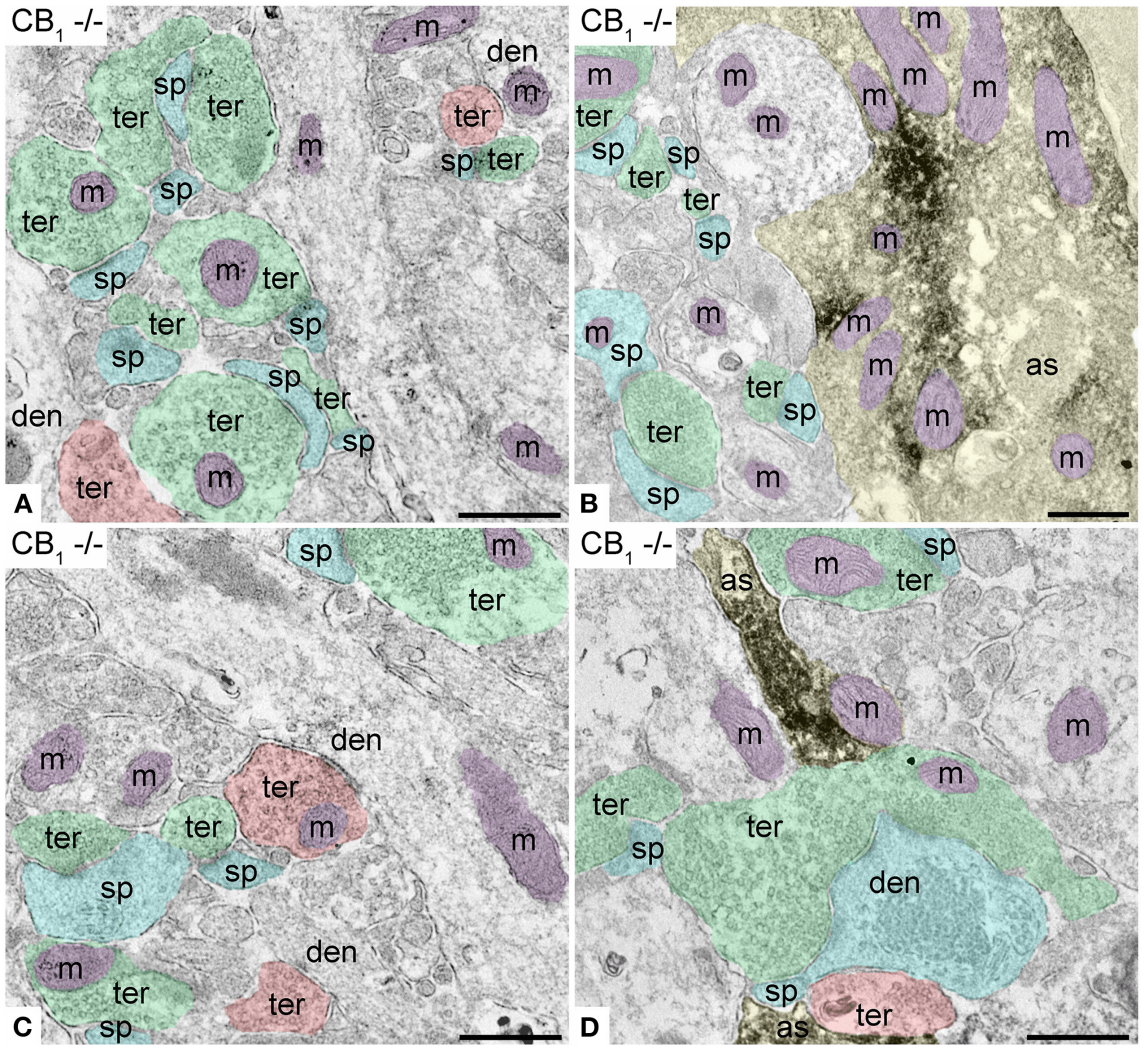
Specificity of the CB_1_R antibodies in dentate ML of the CB_1_R-KO mouse processed for electron microscopy. **(A,C)** Single pre-embedding immunogold method for CB_1_R. **(B,D)** Double pre-embedding immunogold for CB_1_R and immunoperoxidase for GFAP method. No CB_1_R labeling is detected in the outer 2/3 **(A,B)** and inner 1/3 of the layer **(C,D)**. Scale bars: 0.5 μm.

**Figure 4:**
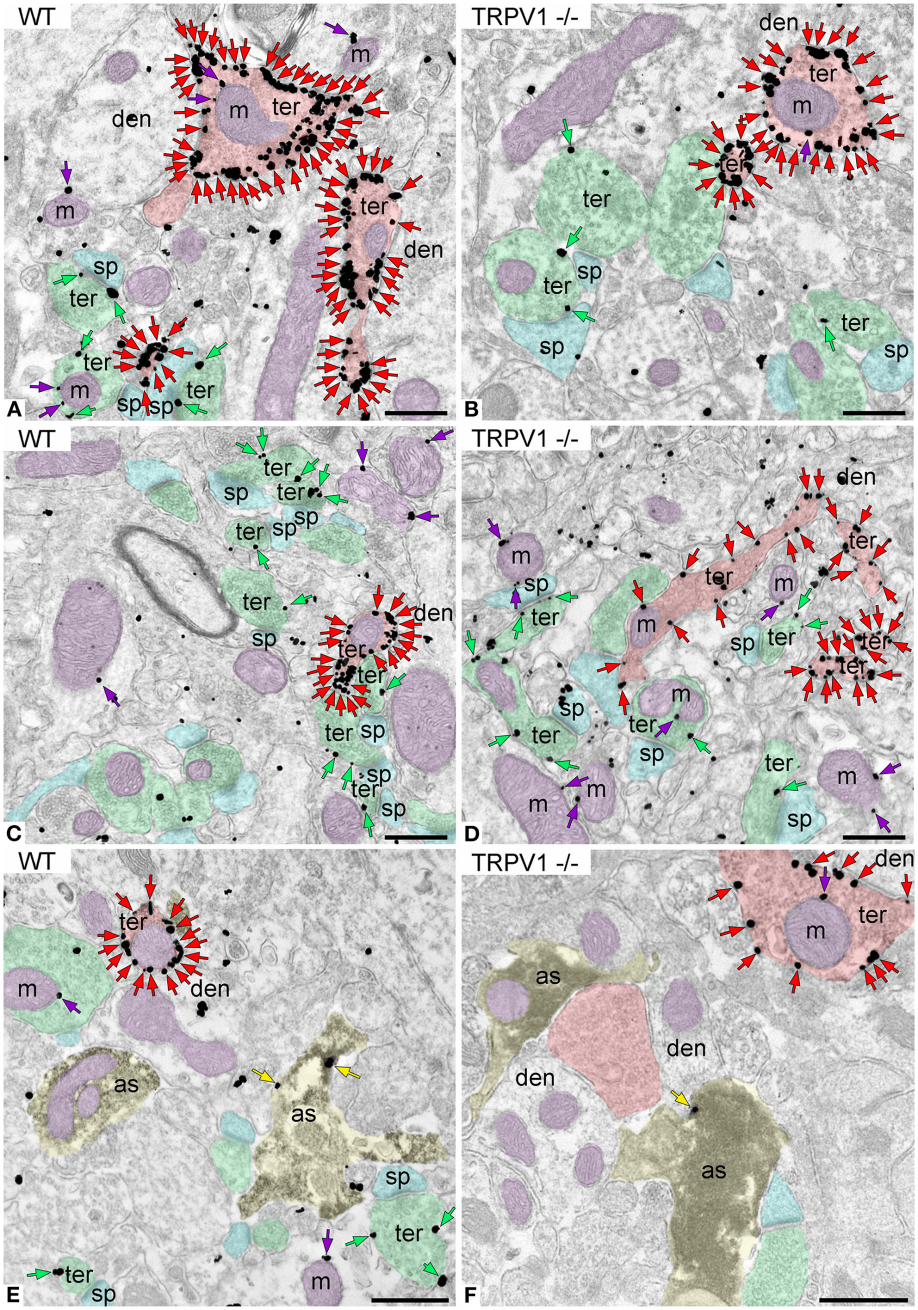
CB_1_R localization in the outer 2/3 ML of WT and TRPV1^−/−^ mice. Pre-embedding immunogold method for electron microscopy. CB_1_R immunoparticles (arrows) are localized to inhibitory terminals (ter, red arrows, red shading), excitatory terminals (ter, green arrows, green shading), and mitochondrial outer membranes (m, purple arrows, purple shading) in WT **(A,C)** and TRPV1^−/−^ **(B,D)**. CB_1_R particles are also on membranes of GFAP positive astrocytic processes (as, yellow arrows, yellow shading) in WT **(E)** and TRPV1^−/−^ **(F)**. Combined pre-embedding immunoperoxidase and immunogold method. sp, dendritic spine; den, dendrite; Scale bars: 0.5 μm.

**Figure 5:**
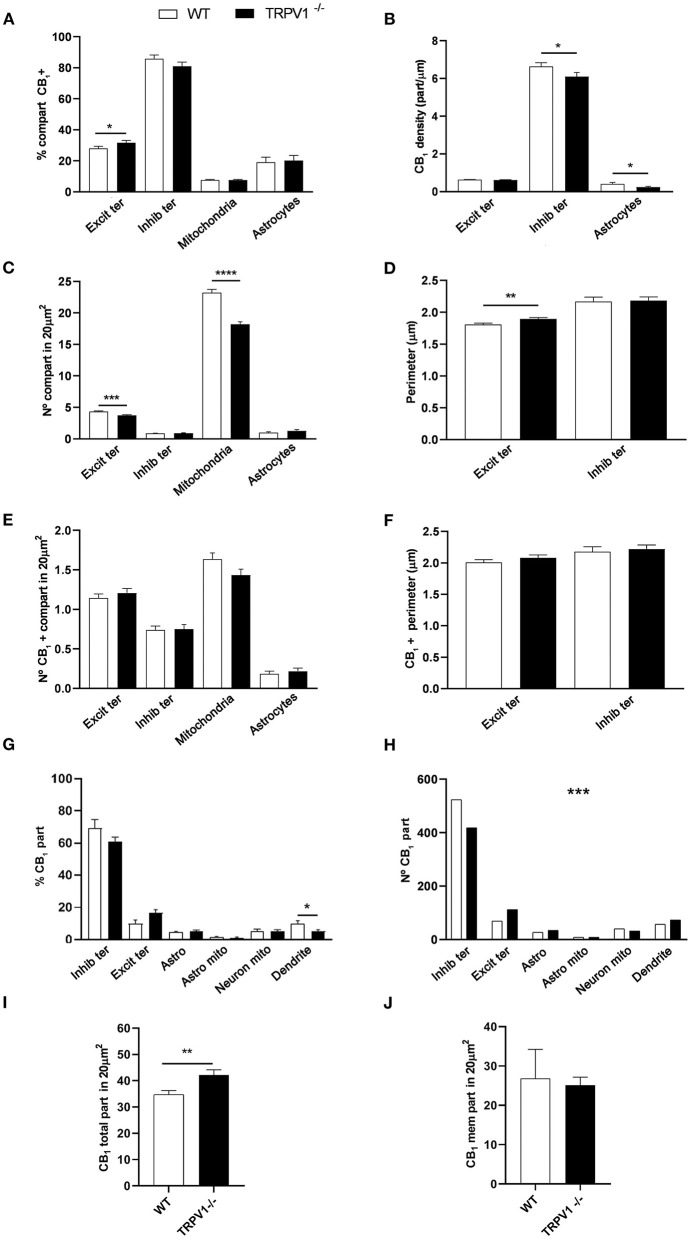
Statistical assessment of CB_1_R labeling in several subcellular compartments in the outer 2/3 ML of WT and TRPV1^−/−^ mice. **(A)** Percentage of CB_1_R positive excitatory and inhibitory terminals, mitochondria, and astrocytes. **(B)** Density of CB_1_R (particles/μm). **(C)** Number of excitatory and inhibitory terminals, mitochondria, and astrocytes in 20 μm^2^. **(D)** Perimeter (μm) of synaptic terminals. **(E)** Number of CB_1_R positive excitatory and inhibitory terminals, mitochondria, and astrocytes in 20 μm^2^. **(F)** Perimeter (μm) of CB_1_R positive synaptic terminals. Data were analyzed by non-parametric or parametric tests (Mann–Whitney *U*-test or Student's *t*-test). **(G)** Proportion of CB_1_R labeling in excitatory and inhibitory terminals, astrocytes, mitochondria (neuronal, astroglial), and dendrites normalized to the total CB_1_R content. **(H)** Number of CB_1_R particles in the compartments analyzed in **(G)**. Chi square test. **(I)** Total number of CB_1_R particles in 20 μm^2^. **(J)** Number of CB_1_R particles found in membranes in 20 μm^2^. Mann–Whitney *U*-test or Student's *t*-test. *p* < 0.05*; *p* < 0.01**; *p* < 0.001***; *p* < 0.0001****. All data are represented as mean ± S.E.M.

First, we confirmed the specificity of the antibody used in this study as CB_1_R labeling was absent in the dentate ML of CB_1_R-KO mice (Figure 3). CB_1_R immunoparticles were localized to excitatory and inhibitory axon terminals, astrocytes, and mitochondria (Figure 4). The proportion of CB_1_R positive excitatory terminals increased in TRPV1^−/−^ (31.71 ± 1.44%; WT: 28.03 ± 1.33%; *p* = 0.0103^*^; Figure 5A) but the receptor density remained unchanged (0.62 ± 0.015 particles/μm; WT: 0.63 ± 0.015 particles/μm; *p* = 0.2772 ns; Figure 5B). Moreover, less excitatory terminals were observed in TRPV1^−/−^ (3.73 ± 0.115; WT: 4.33 ± 0.123 per 20 μm^2^, *p* = 0.0003^***^; Figure 5C) but were of larger size (1.89 ± 0.022 μm, WT 1.80 ± 0.002 μm; *p* = 0.0044^**^; Figure 5D). However, the number of CB_1_R positive excitatory terminals (1.20 ± 0.057; WT: 1.14 ± 0.052 per 20 μm^2^; *p* = 0.4078 ns; Figure 5E) and their perimeter (2.08 ± 0.042 μm; WT: 2.008 ± 0.043 μm; *p* = 0.1431 ns; Figure 5F), were maintained in TRPV1^−/−^.

There were not significant differences in the percentage of CB_1_R positive inhibitory terminals (81 ± 2.70%; WT: 85.79 ± 2.39%; *p* = 0.1594 ns; Figure 5A), number of inhibitory terminals (0.91 ± 0.059; WT: 0.85 ± 0.052 per 20 μm^2^; *p* = 0.7525 ns; Figure 5C), terminal perimeter (2.18 ± 0.055; WT: 2.16 ± 0.071 μm; *p* = 0.846 ns; Figure 5D), number of CB_1_R positive inhibitory terminals (0.75 ± 0.055; WT: 0.74 ± 0.049 per 20 μm^2^; *p* = 0.8098 ns; Figure 5E) or their terminal perimeter (2.22 ± 0.063 μm; WT 2.17 ± 0.079 μm; *p* = 0.1288 ns; Figure 5F). However, CB_1_R particle density significantly decreased in TRPV1^−/−^ inhibitory terminals (6.01 ± 0.214 particles/μm; WT: 6.63 ± 0.199 particles/μm; *p* = 0.0402^*^; Figure 5B).

There were no differences in the proportion of CB_1_R positive mitochondria between TRPV1^−/−^ (7.63 ± 0.40%) and WT (7.68 ± 0.37%; *p* = 0.8748 ns; Figure 5A). However, the number of mitochondria in TRPV1^−/−^ (18.22 ± 0.37 per 20 μm^2^) was significantly lower than in WT (23.24 ± 0.50 per 20 μm^2^; *p* < 0.0001^****^; Figure 5C).

No significant changes were detected neither in the proportion of CB_1_R positive astrocytic profiles in TRPV1^−/−^ (20.19 ± 3.31%) vs. WT (19.1 ± 3.29%; *p* = 0.39 ns; Figure 5A) nor in the number of astrocytic profiles (TRPV1^−/−^: 1.27 ± 0.22 μm^2^; WT: 0.98 ± 0.12 μm^2^ per 20 μm^2^; *p* = 0.2688 ns; Figure 5C). In contrast, a significant decrease in CB_1_R density was observed in TRPV1^−/−^ astrocytes (0.24 ± 0.04 particles/μm; WT: 0.41 ± 0.072 particles/μm; *p* = 0.0452^*^; Figure 5B). Lastly, a reduction in the proportion of CB_1_R particles in inhibitory terminals (TRPV1^−/−^: 61.02 ± 2.65% WT: 69.34 ± 5.28%; *p* = 0.2581 ns) and dendrites (TRPV1^−/−^: 5.02 ± 1.11%; WT: 10.03 ± 1.75%; *p* = 0.0287^*^) and, conversely, an increase in excitatory terminals (TRPV1^−/−^: 16.41 ± 2.25%; WT: 9.84 ± 2.28%; *p* = 0.0573 ns) and astrocytes (TRPV1^−/−^: 5.05 ± 0.87%; WT: 4.41 ± 0.73%; *p* = 0.5856) was observed in TRPV1^−/−^ (Figure 5G). Also, particle distribution changed in TRPV1^−/−^ (Figure 5H), as the number of CB_1_R particles moderately dropped in inhibitory terminals (~21%) but drastically increased in excitatory terminals (~63%). Interestingly, the total number of CB_1_R particles significantly increased in TRPV1^−/−^ outer 2/3 ML (TRPV1^−/−^: 42.1 ± 2.06 particles vs. 34.7 ± 1.55 particles WT per 20 μm^2^; ^**^p = 0.0066; Figure 5I), but the number of membrane particles was not seen to change (TRPV1^−/−^: 25.07 ± 2.06 particles; WT: 26.81 ± 7.4 particles per 20 μm^2^; *p* > 0.05; Figure 5J).

### Cellular and Subcellular Localization of CB_1_R in the Inner 1/3 Dentate ML of TRPV1^−/−^

**Figure 6:**
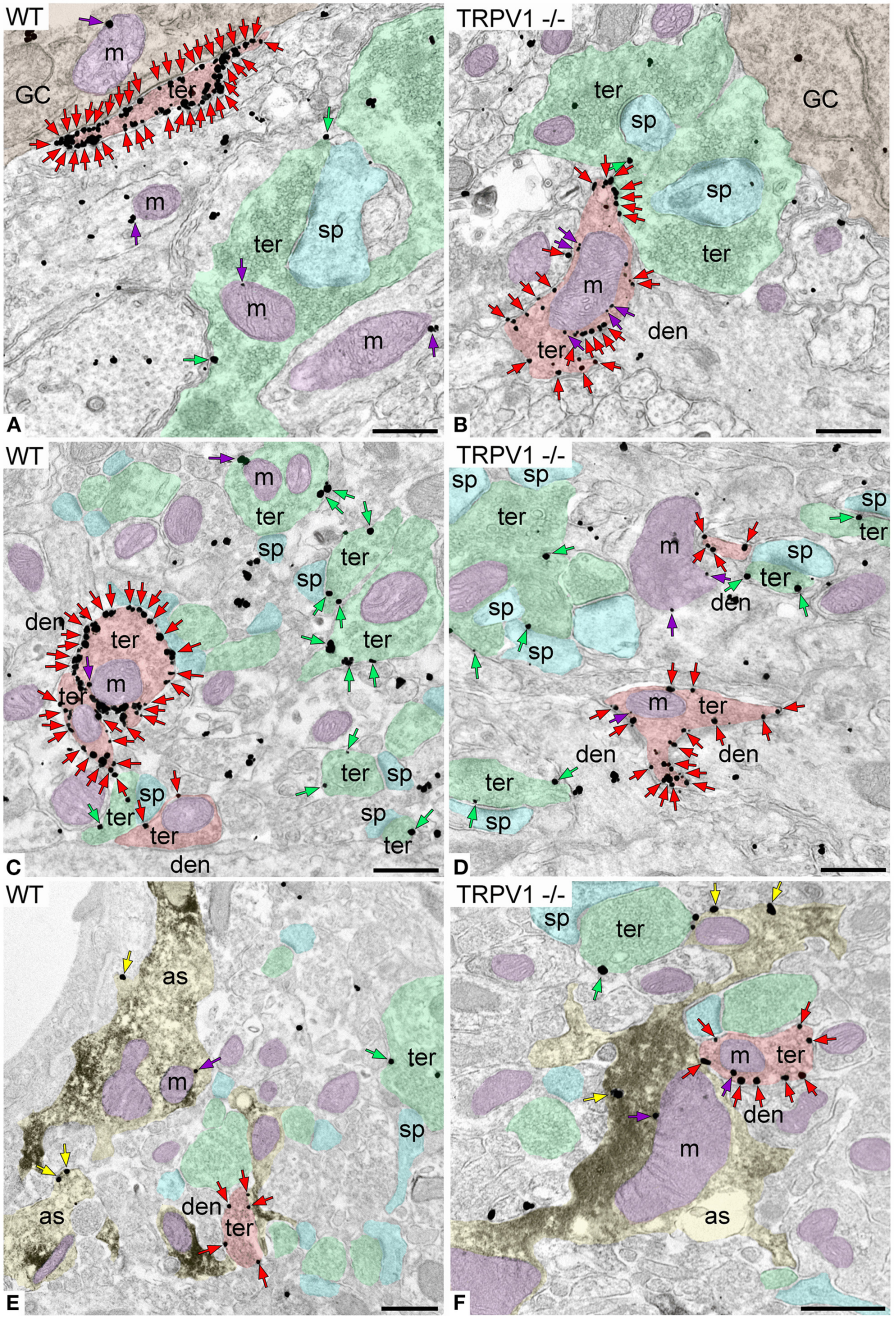
CB_1_R localization in the inner 1/3 ML of WT and TRPV1^−/−^ mice. Pre-embedding immunogold method for electron microscopy. CB_1_R immunoparticles (arrows) are localized to inhibitory terminals (ter, red arrows, red shading), excitatory terminals (ter, green arrows, green shading), and mitochondrial outer membranes (m, purple arrows, purple shading) in WT **(A,C)** and TRPV1^−/−^ **(B,D)**. CB_1_R particles are also on membranes of GFAP positive astrocytic processes (as, yellow arrows, yellow shading) in WT **(E)** and TRPV1^−/−^ **(F)**. Combined pre-embedding immunoperoxidase and immunogold method. sp, dendritic spine; den, dendrite; GC, granule cell. Scale bars: 0.5 μm.

**Figure 7:**
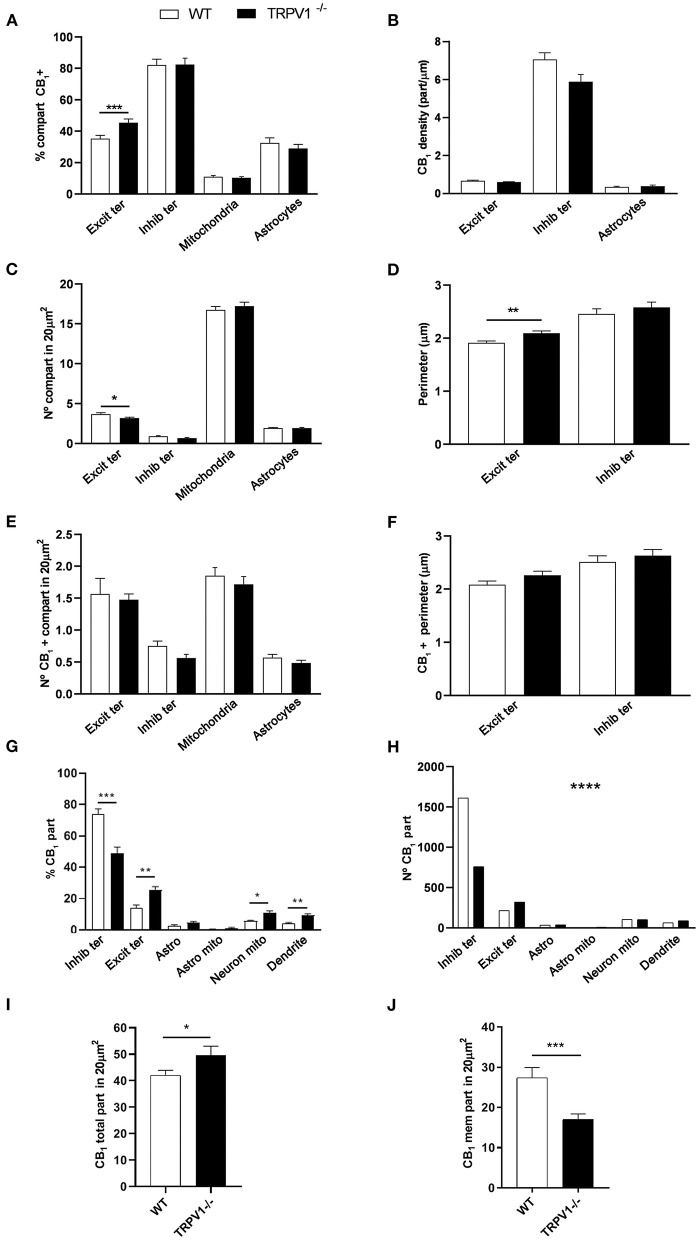
Statistical assessment of CB_1_R labeling in several subcellular compartments in the inner 1/3 ML of WT and TRPV1^−/−^ mice. **(A)** Percentage of CB_1_R positive excitatory and inhibitory terminals, mitochondria and astrocytes. **(B)** Density of CB_1_R (particles/μm). **(C)** Number of excitatory and inhibitory terminals, mitochondria and astrocytes in 20 μm^2^. **(D)** Perimeter (μm) of synaptic terminals. **(E)** Number of CB_1_R positive excitatory and inhibitory terminals, mitochondria and astrocytes in 20 μm^2^. **(F)** Perimeter (μm) of CB_1_R positive synaptic terminals. Data were analyzed by non-parametric or parametric tests (Mann–Whitney *U*-test or Student's *t*-test). **(G)** Proportion of CB_1_R labeling in excitatory and inhibitory terminals, astrocytes, mitochondria (neuronal, astroglial), and dendrites normalized to the total CB_1_R content. **(H)** Number of CB_1_R particles in the compartments analyzed in **(G)**. Chi square test. **(I)** Total number of CB_1_R particles in 20 μm^2^. **(J)** Number of CB_1_R particles found in membranes in 20 μm^2^. Mann–Whitney *U*-test or Student's *t*-test. *p* < 0.05*; *p* < 0.01**; *p* < 0.001***; *p* < 0.0001****. All data are represented as mean ± S.E.M.

CB_1_R immunoparticles were found in excitatory and inhibitory terminals, astrocytes and mitochondria (Figure 6). In TRPV1^−/−^ was observed a significant increase in the proportion of CB_1_R positive excitatory terminals (45.48 ± 2.33%; WT: 35.19 ± 2.20%; *p* = 0.0004^***^; Figure 7A) with no changes in CB_1_R density (0.60 ± 0.019 particles/μm; WT: 0.67 ± 0.025 particles/μm; *p* = 0.0635 ns; Figure 7B), and a reduction in the number of excitatory terminals (3.16 ± 0.13; WT: 3.67 ± 0.16 terminals per 20 μm^2^; *p* = 0.034^*^; Figure 7C). Moreover, the perimeter of excitatory terminals increased in TRPV1^−/−^ (2.09 ± 0.042 μm; WT: 1.91 ± 0.035 μm; *p* = 0.0014^**^; Figure 7D), but the number of CB_1_R positive excitatory terminals (TRPV1^−/−^: 1.47 ± 0.09; WT: 1.56 ± 0.247 per 20 μm^2^; *p* = 0.3029 ns; Figure 7E) and their perimeter (2.26 ± 0.077 μm; WT: 2.08 ± 0.068 μm; *p* = 0.0951 ns; Figure 7F) were not significant different between TRPV1^−/−^ and WT.

There were no changes in TRPV1^−/−^ in the percentage of CB_1_R positive inhibitory terminals (82.43 ± 4.13%; WT: 82.18 ± 3.714%; *p* = 0.8141 ns; Figure 7A), CB_1_R density (5.88 ± 0.37 particles/μm; WT: 7.05 ± 0.35 particles/μm; *p* = 0.0578 ns; Figure 7B), number of inhibitory terminals (0.67 ± 0.063 terminal; WT: 0.87 ± 0.075 terminal per 20 μm^2^; *p* = 0.0892 ns; Figure 7C), their perimeter (2.58 ± 0.101 μm; WT: 2.45 ± 0.096 μm; *p* = 0.2392 ns; Figure 7D), number of CB_1_R positive inhibitory terminals (0.56 ± 0.059 terminal; WT: 0.75 ± 0.073 terminal per 20 μm^2^; *p* = 0.0917 ns; Figure 7E) and their perimeter (2.63 ± 0.113 μm; WT: 2.51 ± 0.115 μm; *p* = 0.3863 ns; Figure 7F).

The overall percentage of CB_1_R positive mitochondria (10.35 ± 0.71%; WT: 11.06 ± 0.73%; *p* = 0.4208 ns; Figure 7A) and the number of mitochondria (17.22 ± 0.48; WT: 16.74 ± 0.43 per 20 μm^2^; *p* = 0.33 ns; Figure 7C) were similar between TRPV1^−/−^ and WT.

**Figure 8:**
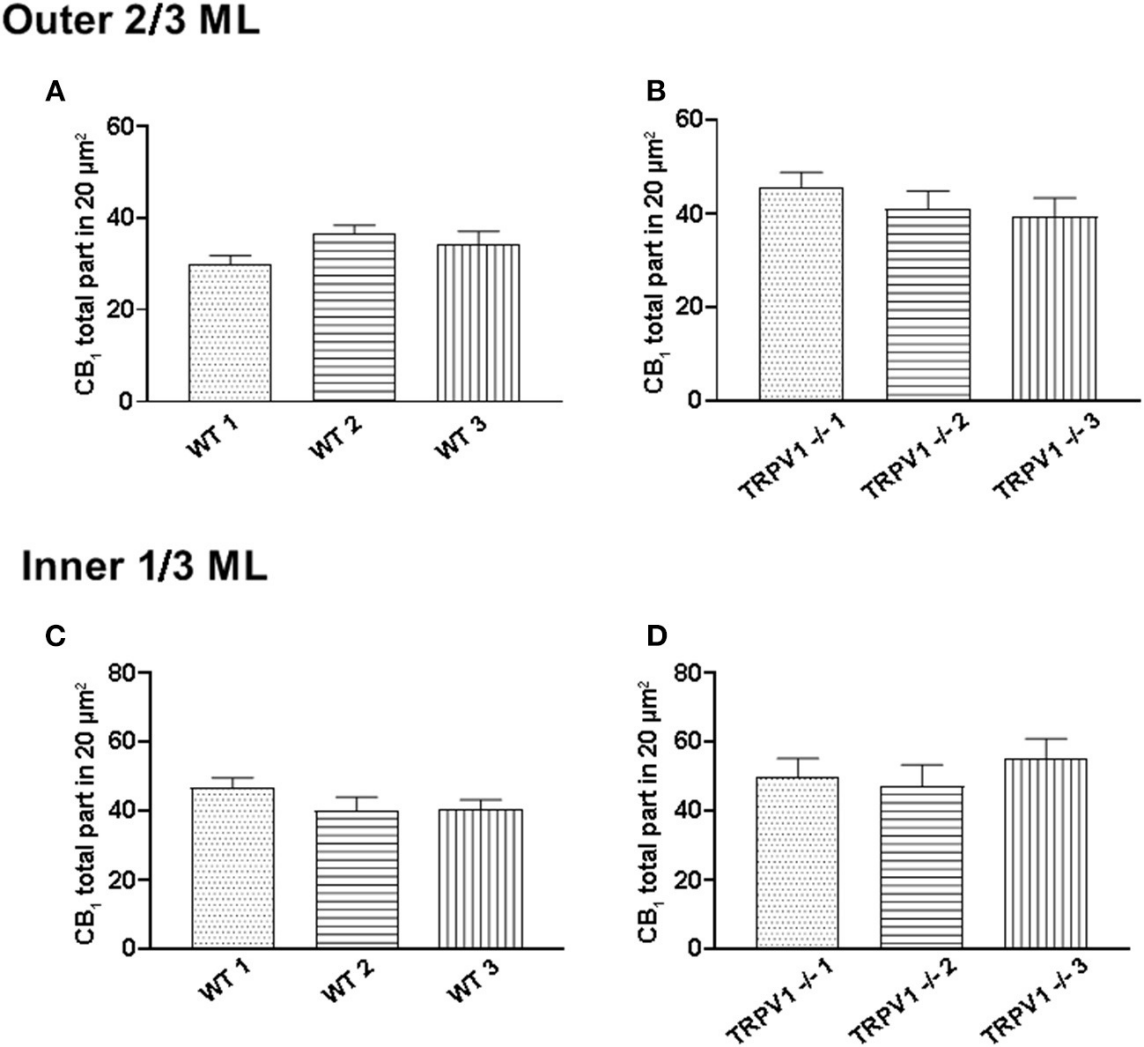
Assessment of sample uniformity in CB_1_R labeling in the outer 2/3 and inner 1/3 ML of WT and TRPV1^−/−^ mice. **(A,C)** The total number of CB_1_R particles per 20 μm^2^ was similar in the 3 WT mice (*p* < 0.05; Kruskal-Wallis test). **(B,D)** The same applied for the 3 TRPV1^−/−^ mice (*p* < 0.05; Kruskal-Wallis).

Also, the proportion of CB_1_R positive astrocytic profiles (28.95 ± 2.67%; WT: 32.53 ± 3.23%; *p* = 0.39 ns; Figure 7A), CB_1_R density (0.39 ± 0.047 particles/μm; WT: 0.34 ± 0.029 particles/μm; *p* = 0.4245 ns; Figure 7B) and the number of astrocytic profiles (1.93 ± 0.072 μm^2^; WT: 1.93 ± 0.082 μm^2^ per 20 μm^2^; *p* = 0.9123 ns; Figure 7C) did not vary between TRPV1^−/−^ and WT. However, the CB_1_R particle distribution seen in WT changed in TRPV1^−/−^ (Figure 7H). Thus, a significant reduction was observed in TRPV1^−/−^in the proportion CB_1_R particles at inhibitory terminals (TRPV1^−/−^: 41.11 ± 3.41%; WT: 63.73 ± 3.24%; *p* < 0.0001^****^), while there was an increase at excitatory terminals (TRPV1^−/−^: 11.28 ± 1.61%; 19.07 ± 1.51%; *p* = 0.0014^**^), neuronal mitochondria (TRPV1^−/−^: 10.76 ± 1.32%; WT: 56.16 ± 0.53%; *p* = 0.0281^*^) and dendrites (TRPV1^−/−^: 9.23 ± 1.14%; WT: 3.92 ± 0.78%; *p* = 0.0017^**^) (Figure 7G). Moreover, a significant decrease in the number of CB_1_R particles was found in inhibitory terminals (~53%) and an increase in excitatory terminals (~50%) in TRPV1^−/−^ (Figure 7H). Finally, a significant increase in the total number of CB_1_R particles was found in TRPV1^−/−^ inner 1/3 ML (49.63 ± 3.39 particles; WT: 42.04 ± 1.87 particles per 20 μm^2^; ^*^p = 0.0475; Figure 7I), while the number of membrane particles decreased significantly in TRPV1^−/−^ vs. WT (TRPV1^−/−^: 16.95 ± 1.37 particles; WT: 27.35 ± 2.64 particles; ^***^p = 0.0002; Figure 7J). The total amount of CB_1_R labeling was similar among the WT mice used (Figures 8A,C). Likewise, labeling was comparable among the TRPV1^−/−^ mice (Figures 8B,D).

## Discussion

The main finding of this study was that the constitutive deletion of the TRPV1 gene impacts on the expression and localization of some elements of the ECS. Thus, the increase in CB_1_R, FAAH, MAGL and NAPE-PLD immunoreactivity in the TRPV1^−/−^ hippocampus indicates the existence of compensatory changes. However, CB_1_R did not change significantly and only FAAH and MAGL increased when measured in western blots of whole hippocampal homogenates. Certainly, this is a powerful technique to detect low protein levels (Garro et al., 2001), but subtle changes in protein expression can be better achieved by homogenate fractioning into P2 extracts and raw synaptosomes (Garro et al., 2001). Thus, MAGL increased in whole TRPV1^−/−^ hippocampal homogenates, but a significant MAGL decrease with no changes in FAAH stood out in TRPV1^−/−^ synaptosomes. One plausible explanation for this discrepancy would be that MAGL expression in astrocytes (Uchigashima et al., 2011) increases in the absence of TRPV1, but decreases the MAGL pool localized in presynaptic terminals (Gulyas et al., 2004; Uchigashima et al., 2011). Finally, 2-AG dysregulation would be expected to occur in TRPV1^−/−^ as DAGLα expression did not change in the absence of TRPV1. FAAH mostly localizes to intracellular organelle membranes but is also on somatic and dendritic membranes (Gulyas et al., 2004), while NAPE-PLD is very highly localized in hippocampal granule cell axons (Egertová et al., 2008). Hence, the FAAH increase in whole homogenates with no obvious changes in NAPE-PLD in TRPV1^−/−^ synaptosomes would drop AEA in TRPV1^−/−^. However, NAPE-PLD immunoreactivity increased in the dentate hilar region and CA3 stratum lucidum, indicating that AEA might be augmented in certain subcellular compartments, e.g., granule cell axons.

Interestingly, CB_1_R expression did not suffer any variation in P2 fractions from TRPV1^−/−^ but significantly decreased in synaptosomal extracts. To circumvent the limiting factors of raw synaptosomes unable to discriminate between presynaptic and postsynaptic compartments, we investigated the CB_1_R localization in TRPV1^−/−^ by high resolution immunoelectron microscopy. We focused on the dentate ML because its outer 2/3 correspond to the termination zone of the glutamatergic entorhino-dentate pathway (Grandes and Streit, 1991) which transmits spatial information through the medial perforant path (Fyhn et al., 2004) and non-spatial information via the lateral perforant path (Burwell, 2000). The inner 1/3 ML receives the glutamatergic mossy cell commissural/associational axons which innervate the dentate granule cells involved in the signaling of environmental and context information (Scharfman and Myers, 2012). Our anatomical data confirmed the biochemical results. Thus, similar changes were found in the inner 1/3 and outer 2/3 ML but with slight differences, that might be reflecting that distinct TRPV1 expression patterns trigger specific compensatory effects. For instance, the relative increase in CB_1_R positive excitatory terminals in TRPV1^−/−^ was more pronounced in the inner 1/3 than outer 2/3 ML. However, taking into account that the CB_1_R density in excitatory terminals did not change throughout the entire ML, the reduction in the number of CB_1_R negative excitatory terminals might likely be explaining the increase seen in CB_1_R positive excitatory terminals. Thus, TRPV1 deletion modifies unevenly the total number of excitatory terminals boosting the proportion of excitatory terminals equipped with CB_1_R, which is in line with the reduced glutamatergic innervation observed in TRPV1^−/−^ hippocampus (Hurtado-Zavala et al., 2017). We observed in TRPV1^−/−^ a decrease in the proportion of CB_1_R particles located in inhibitory terminals in both the inner 1/3 (34.5% ↓) and outer 2/3 ML (15.5% ↓) as well as an increase in CB_1_R labeling in excitatory terminals in the inner 1/3 (83.80% ↑) and outer 2/3 (73.50% ↑) (see below). Furthermore, considering the total number of CB_1_R particles counted, the particles localized in inhibitory terminals decreased ~53% in the inner 1/3 and ~21% in the outer 2/3 ML in TRPV1^−/−^. Nevertheless, the differences in inhibitory terminals in TRPV1^−/−^ relative to WT were minimal because only a small reduction in CB_1_R density was found to be significant in the outer 2/3 ML. Bearing in mind that the majority of CB_1_R particles are localized to GABAergic terminals in the hippocampus (Kano et al., 2009; Gutiérrez-Rodríguez et al., 2017, 2018; Bonilla-Del Río et al., 2019), it would be plausible that an impairment in receptor renewal could be more pronounced in inhibitory than excitatory terminals and therefore easier to be detected. This would also lead to guess that if the overall CB_1_R expression at excitatory terminals remains unchanged, the decrease in CB_1_R density at inhibitory terminals could indeed be responsible for the CB_1_R fall in synaptosomal fractions. Actually, the CRIP1a increase observed in TRPV1^−/−^ might also be playing role as its overexpression interferes with CB_1_R activity and receptor downregulation (Smith et al., 2015).

About 75% of the total TRPV1 positive granule cell dendritic spines and 56% of the dendrites were in the outer 2/3, and the rest in the inner 1/3 ML (Puente et al., 2015). Furthermore, about 30% of the inhibitory synapses in the inner 1/3 ML have TRPV1 mostly localized to postsynaptic dendritic membranes (Canduela et al., 2015). In the absence of TRPV1, CB_1_R may exert a major regulatory effect on the excitatory transmission with important functional consequences in the dentate ML. In this sense, we have recently shown that CB_1_R immunolabeling decreases by 34% in excitatory terminals and the proportion of CB_1_R immunopositive excitatory boutons decreases by 35% in the middle 1/3 ML of the adult mouse subjected to ethanol intake during adolescence (binge drinking model). These deficits in glutamatergic CB_1_Rs were associated with the loss of eCB-eLTD at the MPP-granule cell synapses and an impairment of recognition memory (Peñasco et al., 2020). TRPV1 changes upon the loss or absence of CB_1_R remain to be investigated.

CB_1_Rs are also in mitochondria where they regulate cellular respiration, energy production, and memory formation in the hippocampus (Hebert-Chatelain et al., 2016). The proportion of mitochondrial CB_1_R in TRPV1^−/−^ was maintained regardless TRPV1 is expressed in mitochondrial membranes (Miyake et al., 2015). However, despite that the number of mitochondrial profiles was kept in the inner 1/3 ML, a significant reduction was observed in the outer 2/3 ML, suggesting that TRPV1 could have a direct implication in mitochondrial dynamics. The differences observed between both ML zones may be due to differences in their neuronal composition and distinct effects of TRPV1 absence. Thus, strong TRPV1-mediated mitochondrial calcium-influx causes cytotoxicity and cell death in HEK 293 cells and dorsal root ganglion neurons (Stueber et al., 2017), while TRPV1 knockdown improves mitochondrial function and apoptosis inhibition in primary cardiomyocytes (Sun et al., 2014).

The CB_1_R is also localized to astrocytes (Navarrete and Araque, 2010; Metna-Laurent and Marsicano, 2015; Gutiérrez-Rodríguez et al., 2017). Calcium rise linked to TRPV1 activation drives cytoskeletal rearrangements, microtubule disassembly, and filament reorganization leading to astrocyte migration (Goswami et al., 2007; Morales-Lázaro et al., 2013). However, TRPV1 antagonism has an opposite effect (Ho et al., 2014). We did not detect differences in astrocytic parameters in TRPV1^−/−^, thus astrocytic disturbance does not seem to happen in the absence of TRPV1. TRPV1 deletion could have triggered compensatory mechanisms in other receptors/channels that would replace its function. Furthermore, there were not changes in the proportion of CB_1_R positive astrocytic profiles in TRPV1^−/−^ ML. However, a significant reduction in CB_1_R density in astrocytes was observed in TRPV1^−/−^ outer 2/3 ML resembling the CB_1_R density decrease in astrocytes revealed in a binge-drinking model of ethanol intake (Bonilla-Del Río et al., 2019). Astrocytes participate in inflammatory responses through the release of pro-inflammatory molecules (Farina et al., 2007) that can be soothing by astroglial CB_1_R-mediated mechanisms (Metna-Laurent and Marsicano, 2015). Hence, because of the reduced CB_1_R density in astrocytes, it is reasonable to expect an impairment of an anti-inflammatory response in TRPV1^−/−^. Furthermore, the decrease in astrocytic CB_1_R density could also have functional consequences in synaptic transmission and plasticity, as astrocytes may not be effective in detecting the endocannabinoids produced on demand by neural activity, compromising gliotransmitter availability elicited by cannabinoids at the synapses (Araque et al., 2014).

Altogether, the lack of TRPV1 causes changes in the ECS that might be affecting synaptic transmission and plasticity, and eventually behavior.

## Data Availability Statement

The original contributions presented in the study are included in the article/supplementary material, further inquiries can be directed to the corresponding author/s.

## Ethics Statement

The animal study was reviewed and approved by Committee of Ethics for Animal Welfare of the University of the Basque Country (CEEA/M20/2015/105; CEIAB/M30/2015/106).

## Author Contributions

IE, NP, IG, JS, GG, and PG designed the research. JE-H, IB-D, SG-U, GG, MS-E, and LB-R performed the experimental work and acquired and analyzed the data. JE, IB-D, IE, SG-U, AM, NP, and PG prepared the figures and wrote the manuscript. All authors contributed to the article and approved the submitted version.

## Conflict of Interest

The authors declare that the research was conducted in the absence of any commercial or financial relationships that could be construed as a potential conflict of interest.
